# Combined ventricular output and oxygen delivery are reduced while oxygen extraction fraction is increased in fetuses with Ebstein's Anomaly by MRI

**DOI:** 10.1186/1532-429X-18-S1-O71

**Published:** 2016-01-27

**Authors:** Meng Yuan Zhu, Ioana A Stochitoiu, Edgar Jaeggi, Shi-Joon Yoo, Lars Grosse-Wortmann, Christopher Macgowan, Mike Seed

**Affiliations:** 1 grid.42327.300000000404739646Cardiology, The Hospital for Sick Children, Toronto, ON Canada; 2 grid.17063.33Institute of Medical Science, university of Toronto, Toronto, ON Canada; 3 grid.42327.300000000404739646Physiology & Experimental Medicine, Hospital for Sick Children, Toronto, ON Canada

## Background

Ebstein's anomaly (EA) has variable prognosis. New MRI technology allowing the measurement of vessel blood flow and oxygen content could provide additional prognostic information in the setting of fetal EA.

## Methods

We measured fetal weight, brain weight and lung volume in normal and EA fetuses using MRI. Blood flow and T2 in the major fetal vessels were measured using our previously published technique. Fetal oxygen delivery (DO2), consumption (VO2) and extraction fraction (OEF) were calculated using estimated fetal hemoglobin concentration.

## Results

We studied 30 normal and 8 EA fetuses at 36 weeks gestation. There were 2 deaths in the 4 patients that underwent surgery (all Starnes procedures). We found reduced flows in all measured vessels except ascending aorta (Table 1). There were also significant reduced T2s in ascending aorta and superior vena cava, but no difference in umbilical vein (UV) and descending aorta (Table 1). DO2 was lower, but increases in OEF maintained normal VO2 in EA fetuses (Table 2). Fetal weight and brain weight were lower in EA fetuses, while lung volume was not significantly different (Table 2). Within the EA group, UV flow (P = 0.04), VO2 (P < 0.0001) and lung volume (P = 0.03) were significantly higher in EA newborns not requiring surgery than EA newborn that received surgery.

**Table 1:** MRI measured flow and T2 in major fetal vessels in normal and Ebstein's anomaly fetuses.

	CVO	UV	AAo	SVC	DAo
Normal mean flow (ml/min/kg)	451 ± 56	129 ± 29	205 ± 47	132 ± 31	248 ± 45
Ebstein's mean flow (ml/min/kg)	233 ± 45	83 ± 17	219 ± 40	93 ± 32	135 ± 33
P value	< 0.0001*	< 0.0001*	0.4	0.01*	< 0.0001*
Normal mean T2 (ms)		192 ± 34	119 ± 23	84 ± 13	99 ± 17
Ebstein's mean T2 (ms)		196 ± 26	90 ± 15	74 ± 8	84 ± 22
P value		0.8	0.0005*	0.01*	0.3

CVO: combined ventricular output; UV: umbilical vein; AAo: ascending aorta; SVC: superior vena cava; DAo: descending aorta. - * indicates significantly different result.

**Table 2:** Oxygen consumption, oxygen delivery and oxygen extraction fraction, fetal weight Z score, fetal brain weight Z score and lung volume in normal and Ebstein's anomaly fetuses.

	VO2 (ml/min/kg)	DO2 (ml/min/kg)	OEF	Fetal weight Z score	Brain weight Z score	Lung volume
Normal	7.0 ± 1.9	20.1 ± 5.1	0.35 ± 0.07	0.2 ± 0.6	0.1 ± 0.9	64 ± 24
Ebstein	6.3 ± 1.3	13.4 ± 2.5	0.47 ± 0.09	-0.3 ± 0.5	-1.3 ± 0.7	55 ± 17
P value	0.3	0.008*	0.01*	0.03*	0.0003*	0.3

VO2: oxygen consumption; DO2: oxygen delivery; OEF: oxygen extraction fraction. - * indicates significantly different result.

## Conclusions

MRI revealed a ~50% reduction in CVO in EA fetuses compared to normal fetuses, which is greater than any other type of CHD. While EA subjects had similar placental function (indicated by similar UV T2), lower CVO resulted in reduced UV flow, therefore decreased DO2. EA fetuses had increased OEF compensating for the reduced DO2, but body and brain development were still reduced. In addition, MRI-based lung volume measurements, UV flow and VO2 maybe associated with postnatal outcome in EA fetuses. Therefore, with more experience, the MRI technique may provide useful prognostic information in Ebstein's anomaly pregnancies.

